# Supplementary material for: Hot Spot Mutagenesis Improves the Functional Expression of Unique Mammalian Odorant Receptors
Source: Int J Mol Sci. 2021 Dec 28;23(1):277. doi: 10.3390/ijms23010277 (PMC8745346; doi:10.3390/ijms23010277)
Supplement: Supplementary file 1 [file ijms-23-00277-s001.zip › Supporting Information_ijms-1494812_R1.pdf]

Supporting Information

Hot spot mutagenesis improves the functional expression of unique mammalian odorant receptors.

Yosuke Fukutani[1,2,3], Yuko Nakamura[1], Shunta Miyanaga[1], Kentaro Ikegami[1,2], Nonoko Muto[1], Reina Kanemaki[1], Keiichi Noguchi[4], Ikuroh Ohsawa[5], Hiroaki Matsunami[2,3,6], Masafumi Yohda[1,3]

[1]Department of Biotechnology and Life Science, Tokyo University of Agriculture and Technology, Koganei, Tokyo 184-8588, Japan; [2]Department of Molecular Genetics and Microbiology, Duke University Medical Center, Durham, NC 27710, United States; [3]Institute of Global Innovation Research, Tokyo University of Agriculture and Technology, Koganei, Tokyo 184-8588, Japan; [4]Instrumentation Analysis Center, Tokyo University of Agriculture and Technology, Koganei, Tokyo, 184-8588, Japan. [5]Biological Process of Aging, Tokyo Metropolitan Institute of Gerontology, Tokyo, 173-0015, Japan, [6]Department of Neurobiology, Duke Institute for Brain Sciences, Duke University Medical Center, Durham, NC 27705, United States;

TABLE OF CONTENTS

Figure S1 Normalized value of the cell-surface expression of the Rho-tagged ORs and each mutants.

Figure S2 Cell-surface expression of the Rho-tagged RTP independent ORs and mutants.

Figure S3 Cell-surface expression of the Rho-tagged RTP dependent ORs and mutants.

Figure S4 Comparison of amino acid sequences of Ofr544 and its homologues

Figure S5 Analysis of Olfr544 D3,39 mutants

Figure S6 Effect of 3.39E and 3.43L mutant for Human ORs

Figure S7 Sequence conservation of the 15 residues predicted the sodium ion binding pocket in mouse and human ORs

	Olfr78
WT	1.000
4.53G	0.339
6.48Y	1.081
7.40F	0.708
7.41Y	1.127
C-term(Q297)E	1.750

	Olfr168
WT	1.000
3.52Y	1.315
IC2(R134)I	0.745
7.35K	0.975
7.40F	0.975

	Olfr194
WT	1.000
IC2(L130)H	3.562
EC2(T166)P	0.874

	Olfr204
WT	1.000
IC2(L130)H	1.120
EC2(T166)P	0.884

	Olfr539
WT	1.000
IC2(R144)I	1.084
7.40F	1.000

	Olfr544
WT	1.000
3.39E	8.643
4.50W	0.636
4.53G	0.460
EC2(V170)L	0.604
EC2(R171)P	0.652
7.35K	0.568

	Olfr644
WT	1.000
4.53G	0.793
5.46I	0.800
7.35K	0.517
7.40F	1.294

	Olfr666
WT	1.000
4.53G	0.907
7.35K	0.907
7.40F	0.907
C-term(Q301)E	1.150

	Olfr733
WT	1.000
IC2(Y131)H	1.191
7.41Y	1.009

	Olfr1057
WT	1.000
IC2(L128)H	1.043
4.53G	0.940
EC2(V166)L	1.053

	Olfr1226
WT	1.000
7.41Y	0.523

	Olfr1364
WT	1.000
3.52Y	1.009

	Olfr1336
WT	1.000
7.40F	0.828

	Olfr1508
WT	1.000
IC2(Q134)H	0.789
6.48Y	0.671
7.41Y	0.482

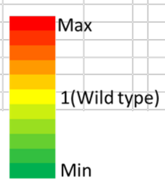

	Olfr124
WT	1.000
3.51Y	0.912
EC2(164)P	1.106
5.46I	0.978
5.46I	0.978
7.36V	0.987
C-term(293)D	0.887
C-term(300)K	1.076
C-term(301)L	1.024

	Olfr167
WT	1.000
4.50W	1.078
4.53F	0.922
5.49V	1.079
6.52I	0.964
C-term(300)K	1.000
C-term(301)L	0.951

	Olfr211
WT	1.000
2.58V	0.956
IC2(132)V	0.935
4.53G	0.991
4.53F	0.894
5.46I	1.059
7.36V	0.984
7.39F	0.913

	Olfr476
WT	1.000
2.58V	0.966
IC2(128)H	0.973
IC2(132)V	0.986
4.50W	1.005
4.53F	1.005
6.52I	0.977
C-term(293)D	0.972
C-term(301)L	0.981

	Olfr491
WT	1.000
2.58V	1.061
IC2(128)H	1.041
IC2(132)V	1.061
4.53F	1.020
6.52I	1.020
7.34K	1.025
C-term(293)D	0.954
C-term(301)L	0.934

	Olfr502
WT	1.000
2.58V	0.968
3.51Y	0.950
IC2(128)H	0.886
IC2(132)V	0.959
4.50W	0.941
EC2(164)P	0.904
6.52I	0.954
C-term(300)K	0.932
C-term(301)L	0.904

	Olfr919
WT	1.000
2.58V	0.913
IC2(128)H	0.977
IC2(132)V	1.121
4.50W	1.009
EC2(164)P	0.853
5.46I	1.111
6.47Y	0.969
6.52I	0.981
7.36V	1.066
7.42V	0.872
C-term(293)D	1.111

	Olfr982
WT	1.000
3.43L	1.536
4.53F	0.888
EC2(164)P	0.727
5.46I	1.253
5.49V	0.808
6.31K	1.000
7.34K	0.971
7.36V	1.056
C-term(300)K	0.972

	Olfr992
WT	1.000
2.58V	1.163
3.39E	1.371
4.50W	1.138
EC2(163)L	0.991
EC2(164)P	0.997
5.46I	1.015
5.49V	1.064
6.52I	1.230
7.36V	1.138
C-term(300)K	1.052

	Olfr1093
WT	1.000
2.58V	0.883
3.55C	1.017
IC2(128)H	0.839
4.50W	1.007
4.53F	1.057
EC2(191)V	0.899
6.52I	0.832
7.34K	0.785
C-term(293)D	0.762

	Olfr1484
WT	1.000
2.58V	1.031
3.51Y	1.122
4.50W	0.866
5.46I	1.049
5.49V	0.977
7.36V	0.996

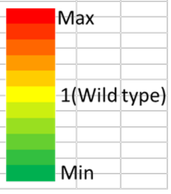

Supplementary Figure S1 Normalized value of the cell-surface expression of the Rho-tagged ORs and each mutants.

Refer to the Geometric mean value calculated by FlowJo software, the values of OR mutants were used as the wild type (=1).

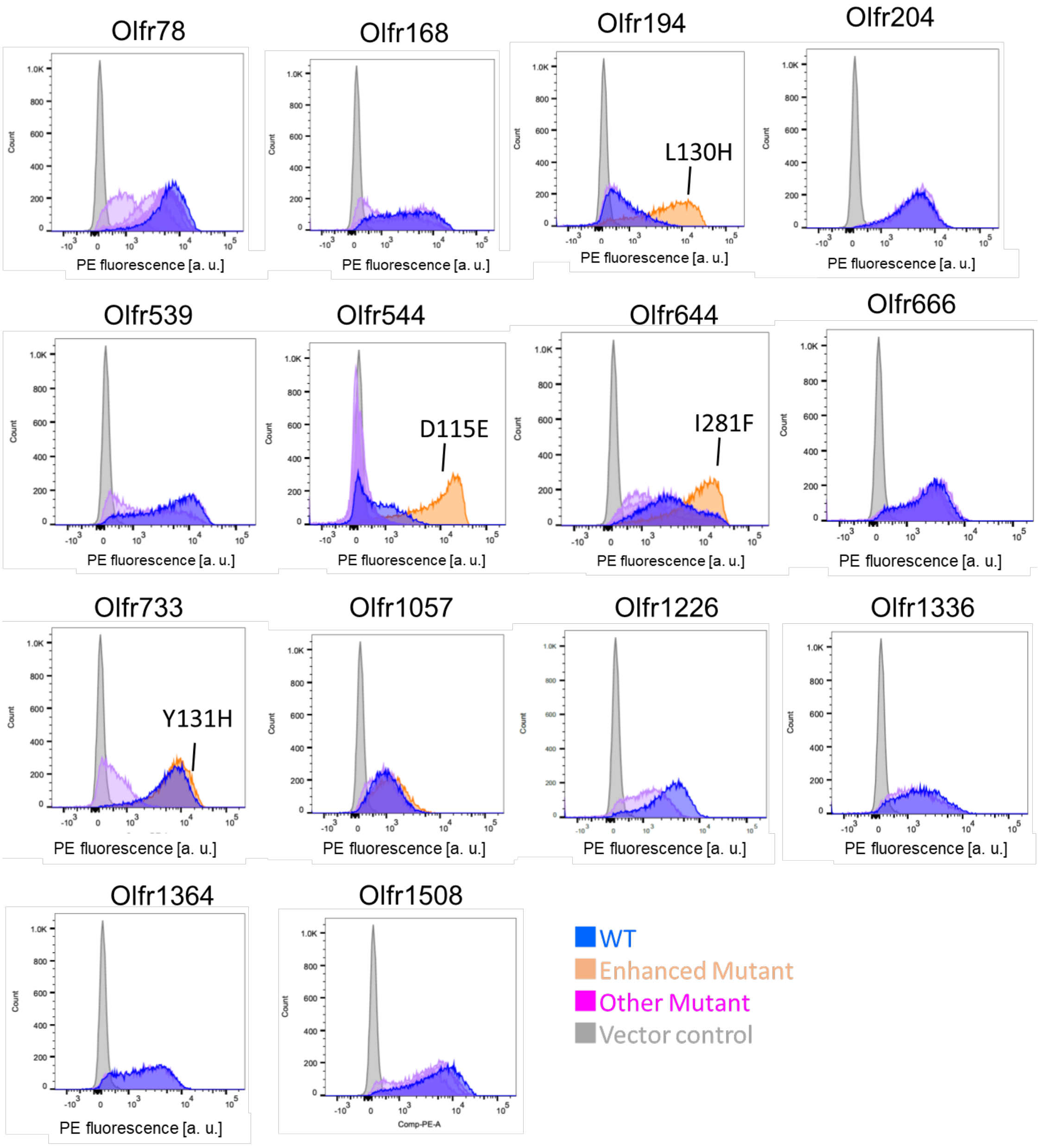

Supplementary Figure S2 Cell-surface expression of the Rho-tagged RTP independent ORs and each mutants.

Each mutant was transfected into HEK293T cells, and the cell surface expression level was measured.

X-axis: PE fluorescence, Y-axis: cell number.

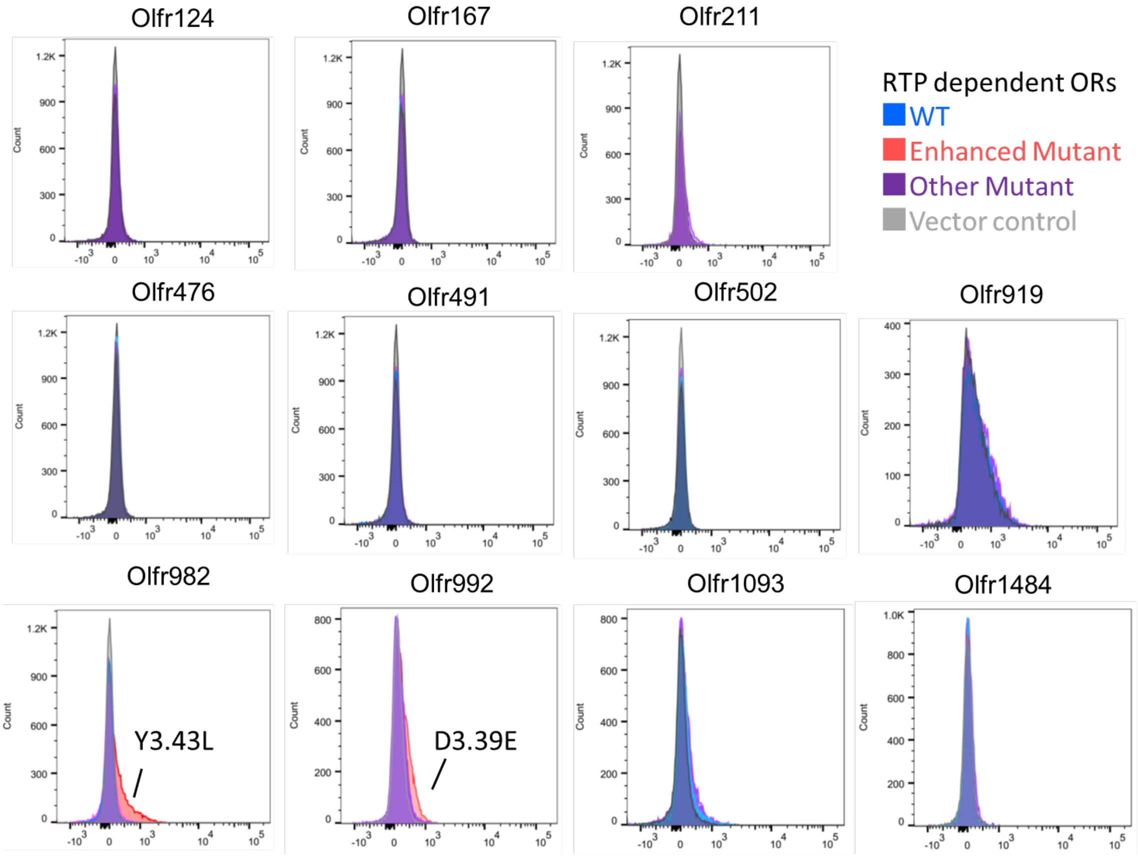

Supplementary Figure S3 Cell-surface expression of the Rho-tagged RTP dependent ORs and each mutants.

Each mutant was transfected into HEK293T cells, and the cell surface expression level was measured.

X-axis: PE fluorescence, Y-axis: cell number.

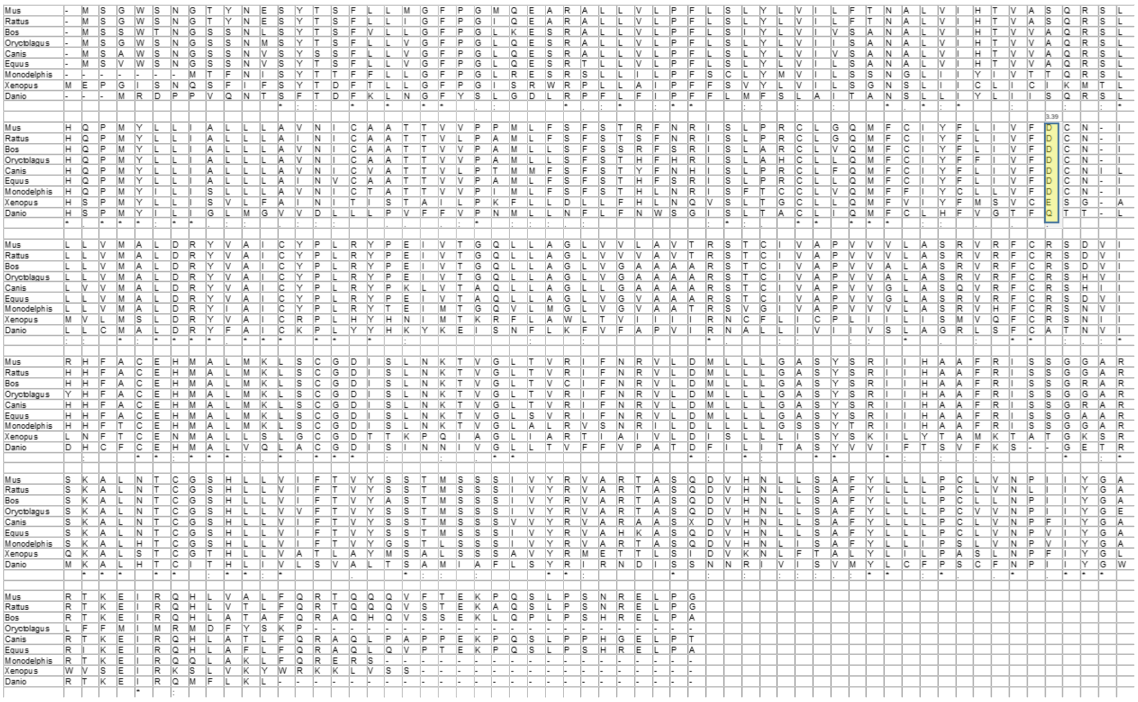

Supplementary Figure S4 Comparison of amino acid sequences of Ofr544 and its homologues

Amino acid sequences of mouse Olfr544 from 9 species were aligned by ClustalW. The position of D115$^{BW3.39}$ was marked as yellow.

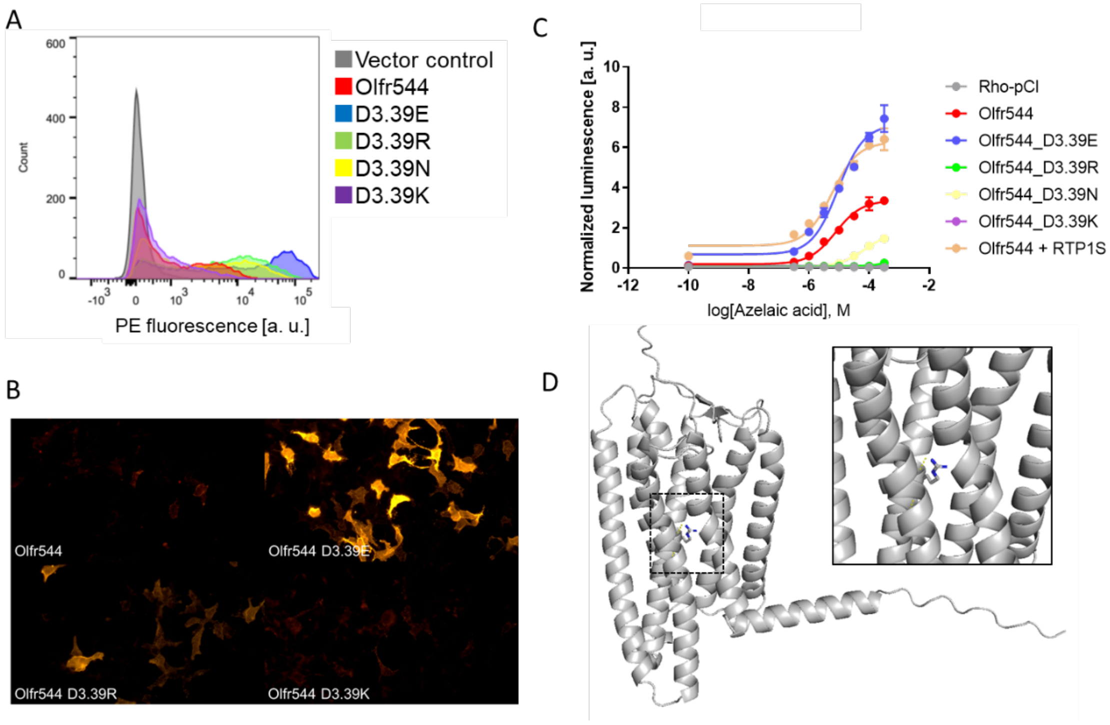

Supplementary Figure S5 Analysis of Olfr544 D115$^{BW3.39}$ mutants

A) The cell-surface expression of the Rho-tagged ORs and D3.39 mutants. Each mutant was transfected into HEK293T cells, and the cell surface expression level was measured. X-axis: PE fluorescence, Y-axis: cell number. B) Immunocytochemical image of Olfr544 and mutants stained by anti-Rho4D2 mouse antibody and PE-conjugated anti-mouse IgG goat antibody. C) Dose response curve of Olfr544 and D3.39 mutants to nonanedioic acid. Error bars indicate s.e.m (n =3). D) Structural model of Olfr544 and D3.39R mutant using Alphafold 2 prediction.

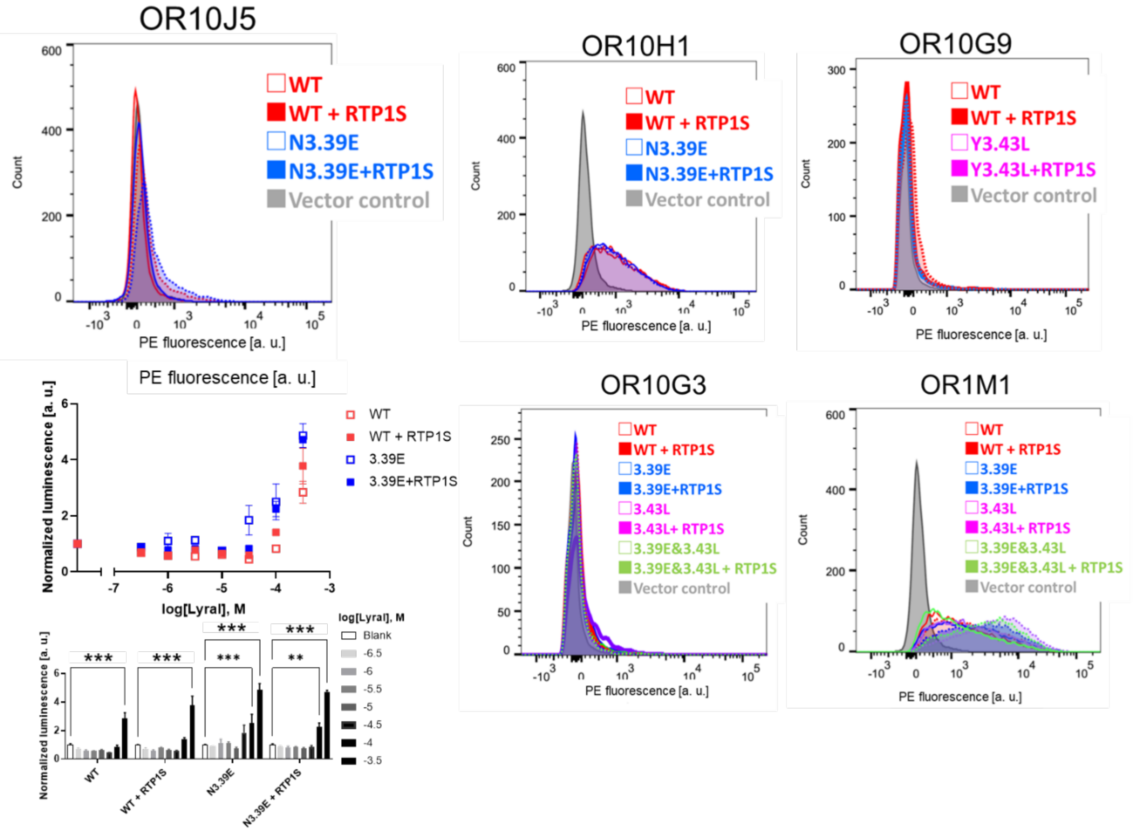

Supplementary Figure S6 Effect of 3.39E and 3.43L mutant for Human ORs

Left) Analysis of OR10J5. Top：The cell-surface expression of the Rho-tagged ORs and D3.39 mutants. Each mutant was transfected into HEK293T cells, and the cell surface expression level was measured. X-axis: PE fluorescence, Y-axis: cell number. Bottom: Dose response curve of OR10J5 to Lyral. Error bars indicate s.e.m (n =3). Multiple comparisons were performed using one-way ANOVA followed by Dunnett's test (**p<0.01, ***p<0.001)

Right) The cell-surface expression of the Rho-tagged ORs (OR10H1, OR10G3, OR10G9 an OR1M1) and 3.39E and 3.43L mutants.

Mouse

	1.50	1.53	2.46	2.47	2.49	2.50	3.39	3.43	6.44	6.48	7.45	7.46	7.49	7.50	7.53
A	0	0	0	252	54	0	0	0	8	0	65	14	0	1	0
C	0	0	0	0	16	0	0	1	0	3	0	3	0	0	0
D	0	0	0	2	0	917	121	0	0	0	0	0	2	0	0
E	0	0	0	0	0	169	933	0	0	0	3	0	0	0	0
F	0	0	83	1	7	0	0	34	15	269	10	0	0	0	10
G	0	0	0	4	1	0	5	0	0	0	13	1	0	0	0
H	0	0	0	0	0	0	4	0	0	23	0	0	0	1	1
I	0	655	1	0	189	0	1	8	116	4	237	0	0	0	0
K	0	1	1	0	0	0	3	0	0	0	0	0	1	0	0
L	0	210	1002	3	323	0	2	1020	7	21	2	1	0	2	0
M	0	179	4	1	76	0	2	8	6	0	2	0	0	0	0
N	1093	0	0	0	20	7	10	0	0	0	17	0	1083	0	0
P	0	0	0	0	1	0	0	1	0	0	160	1050	0	1087	0
Q	0	0	0	0	0	0	7	0	0	14	0	12	0	0	0
R	0	0	0	0	0	0	1	0	0	0	0	0	0	0	0
S	0	0	0	829	65	0	0	0	0	5	47	11	6	2	0
T	0	22	0	0	106	0	1	0	3	0	466	1	0	0	0
V	0	26	1	1	235	0	3	1	938	1	71	0	0	0	0
W	0	0	0	0	0	0	0	0	0	0	0	0	0	0	0
Y	0	0	1	0	0	0	0	20	0	753	0	0	1	0	1082
Total	1093	1093	1093	1093	1093	1093	1093	1093	1093	1093	1093	1093	1093	1093	1093
OR consensus	N	I	L	S	L	D	E	L	V	Y	T	P	N	P	Y
Other GPCRs	N	V	L	A	A	D	S	L	F	W	N	S	N	P	Y

Human

	1.50	1.53	2.46	2.47	2.49	2.50	3.39	3.43	6.44	6.48	7.45	7.46	7.49	7.50	7.53
A	0	0	0	87	21	0	0	1	3	0	17	4	0	0	0
C	0	0	0	1	1	0	0	0	0	4	0	0	0	1	3
D	0	0	0	2	0	318	51	0	0	0	0	0	0	0	1
E	0	0	0	0	0	67	315	0	0	0	0	0	0	0	0
F	0	0	22	1	2	0	0	11	8	92	7	0	0	0	9
G	0	0	0	2	0	1	2	0	0	0	1	0	0	0	0
H	0	0	0	0	0	0	7	0	0	10	0	1	0	0	2
I	0	242	0	0	72	0	0	7	46	1	57	0	0	0	0
K	2	2	1	0	0	0	1	0	0	0	0	0	1	0	0
L	0	82	364	0	121	0	0	361	4	6	1	3	0	4	2
M	0	59	3	0	35	0	1	1	4	0	1	0	0	0	0
N	386	0	0	0	2	3	7	0	0	0	15	0	377	0	0
P	0	0	0	1	1	0	0	1	0	0	63	371	2	378	0
Q	0	0	0	0	0	0	3	0	0	2	0	3	1	0	1
R	0	0	0	0	2	0	0	0	0	1	0	1	0	0	0
S	0	0	0	295	24	0	2	0	0	5	14	5	8	7	0
T	1	1	0	0	34	0	1	0	0	2	198	2	1	0	0
V	0	4	0	1	75	0	0	0	325	0	16	0	0	0	0
W	0	0	0	0	0	0	0	0	0	0	0	0	0	0	0
Y	1	0	0	0	0	1	0	8	0	267	0	0	0	0	372
Total	390	390	390	390	390	390	390	390	390	390	390	390	390	390	390
OR consensus	N	I	L	S	L	D	E	L	V	Y	T	P	N	P	Y
Other GPCRs	N	V	L	A	A	D	S	L	F	W	N	S	N	P	Y

Supplementary Figure S7 Amino acids conservation in mouse and human ORs on the 15 residues placed surrounding the sodium ion binding pocket among class A GPCR
